# Transplantation tolerance: lessons from experimental rodent models

**DOI:** 10.1111/j.1432-2277.2007.00533.x

**Published:** 2007-10

**Authors:** Cherry I Kingsley, Satish N Nadig, Kathryn J Wood

**Affiliations:** Transplantation Research Immunology Group, Nuffield Department of Surgery, John Radcliffe Hospital, University of Oxford Oxford, UK

**Keywords:** central tolerance, peripheral tolerance, rodent models, transplantation

## Abstract

Immunological tolerance or functional unresponsiveness to a transplant is arguably the only approach that is likely to provide long-term graft survival without the problems associated with life-long global immunosuppression. Over the past 50 years, rodent models have become an invaluable tool for elucidating the mechanisms of tolerance to alloantigens. Importantly, rodent models can be adapted to ensure that they reflect more accurately the immune status of human transplant recipients. More recently, the development of genetically modified mice has enabled specific insights into the cellular and molecular mechanisms that play a key role in both the induction and maintenance of tolerance to be obtained and more complex questions to be addressed. This review highlights strategies designed to induce alloantigen specific immunological unresponsiveness leading to transplantation tolerance that have been developed through the use of experimental models.

## Introduction

Classic experiments reported by Billingham, Brent and Medawar more than 50 years ago [1] demonstrated that skin grafts from major histocompatibility complex (MHC) disparate donor mice would be accepted indefinitely when recipient mice had been exposed to donor alloantigen in the neonatal period. These findings set the stage for the use of experimental animal models to be used in the quest to achieve transplantation tolerance. As these studies were reported, organ transplantation has evolved from an experimental therapy to the mainstream treatment option for established organ failure. The previous hurdle of acute rejection has been better controlled with the development of newer, more potent, immunosuppressive medications and regimens where combinations of immunosuppressive drugs are used. The achievements of improved immunosuppression have, however, permitted the deleterious sequelae of the long-term use of immunosuppressive agents as well as chronic transplant dysfunction to become leading causes of recipient morbidity and in some cases mortality as well as organ failure in long-term transplant recipients [2,3]. Thus, although the technique of transplantation has improved and pharmacotherapeutics to limit acute rejection episodes have evolved, the harmful ramifications of nonspecific immunosuppression still persist, mandating the need for tolerance induction therapies.

Experimental models have proven extremely useful for the field of transplantation to progress to its current state; however, it is necessary to revisit these models to investigate methods of tolerance induction in situations that are more relevant to the clinical setting. This review discusses various methods utilized in experimental models to achieve alloantigen-specific immunologic unresponsiveness as well as the future direction of experimental models in the field of transplantation.

## Central transplantation tolerance

It is well known that the thymus is essential for both T-cell maturation and the induction of tolerance to self antigens. During maturation, thymocytes are positively selected provided that their TCR has a ‘low’ intrinsic affinity for self MHC. The process of negative selection results in the elimination of thymocytes that react to self-MHC+ peptide with too high an avidity. The aim of these two processes is to ensure that mature T cells emigrating from the thymus will be able to recognize and respond to peptides derived from the external environment bound to self MHC molecules when they are presented in the periphery but unable to respond to self peptides presented by MHC molecules of the host. Two of the ways in which this natural process of deletion of self reactive T cells has been exploited to try and induce tolerance to donor alloantigens are described below.

### Intrathymic administration of antigen

One approach to achieve central deletion of donor antigen reactive T cells has involved intrathymic administration of alloantigen. Studies using this approach were initiated in the 1960s and developed further by Posselt *et al.* who confirmed that the thymus was a suitable transplant site. This study, in the rat model, demonstrated the principle that intrathymic injection of allogeneic islets together with lymphocyte depletion in the periphery reversed diabetes and induced normoglycaemia [4]. Limiting dilution analysis to determine the frequency of donor alloantigen reactive cells remaining in the periphery after intrathymic injection of the islets suggested that, indeed, deletion of donor alloantigen reactive T cells had occurred. This supposition was confirmed by a study using a TCR transgenic (Tg) model that demonstrated directly that the deletion of donor reactive thymocytes after intrathymic injection of donor leukocytes results in the induction of operational tolerance [5]. Since this observation, many other studies have confirmed that intrathymic injection of donor antigen or allopeptides along with peripheral leukocyte depletion may lead to the successful induction of operational donor-specific tolerance in rodent models [6–8]; however, the feasibility of this approach in larger species is still questionable. Furthermore, after the intrathymic delivery of allopeptide, donor antigen persists in the thymus for only a defined period. Therefore, intrathymic delivery of donor antigen, in contrast to establishment of a stable mixed chimaera (see below), provides a transient presence of donor derived antigen and stimulation of tolerant mechanisms, rather than generating persistent deletion of thymocytes. Therefore, additional strategies are needed to control alloreactive T cells, after the intrathymic delivery of alloantigen, to transplant a solid-organ graft in the long term [6].

In a clinical study, Remuzzi *et al.* investigated the safety and tolerability of an intrathymic injection of donor splenocytes peri-operatively [9]. Preliminary results showed that although intrathymic injection did not have any adverse consequences for the two patients who consented to participate in this pilot study, this procedure did not prevent acute cardiac allograft rejection. The authors attributed this failure to prevent graft rejection to the simultaneous use of immunosuppressive agents, suggesting that specific conditions need to be optimized before protocols involving intrathymic cellular administration can be clinically exploited safely and effectively in the future. More information about the potential impact of the simultaneous administration of immunosuppressive drugs on the efficacy of intrathymic delivery of alloantigen would be essential for future studies.

### Mixed chimaerism

Early work by Sachs *et al.* revealed that irradiated mice reconstituted with a mixture of T-cell depleted host and donor bone marrow accepted donor skin grafts permanently, rejected third party grafts and did not develop graft versus host disease (GVHD) [10]. The success of this experimental approach relied on the generation of stable mixed chimaerism, a state in which donor and host haematopoietic elements from multiple lineages coexist. These and other studies showed that once host T cells are sufficiently ablated to enable bone marrow engraftment to be achieved, tolerance to fully MHC mismatched grafts can be attained [11].

The requirement for pretransplant host conditioning with sub-lethal irradiation and/or myeloablative agents have limited the development and clinical application of this approach to its fullest extent. Nevertheless, data from rodent as well a large animal studies and more recently clinical studies demonstrate that mixed chimaerism is an effective approach for inducing tolerance to a defined set of donor alloantigens [12–17]. To progress this approach, much work in rodent models has focused on replacing these toxic therapies with less harmful protocols that reduce host morbidity and have greater clinical potential. Alternate approaches to myeloablative therapy were pursued in mouse experimental models wherein the concomitant infusion of high-dose bone marrow with nonmyeloablative regimens promoted the deletion of donor reactive cells in the thymus [18–21]. Co stimulatory blockade has been reported to eliminate the need for cytoreduction and provide long-term graft survival across multiple organ systems in experimental models [14,22–24]. In large animal models, T-cell depletion has also been shown to be effective in producing stable mixed chimaerism [25]. Early experimental evidence suggesting that full chimaeras may reject donor grafts, a phenomenon known as ‘split tolerance’, may also apply to the condition of stable mixed chimaerism unmatched for minor antigens [26,27]. Although feasible in experimental models, matching of minor antigens may not be possible in routine clinical practice. Therefore, it is necessary to overcome the obstacle of ‘split tolerance’ before further strategies utilizing nonmyeloablative conditioned mixed chimaerism can be translated to the clinic [26].

An elegant study by Wekerle *et al.* revealed that lasting chimaerism and donor specific transplantation tolerance could be achieved through a protocol, which combined the administration of a high dose of fully MHC mismatched bone marrow with a single injection of anti-CD40L and cytotoxic T-lymphocyte associated antigen 4-Ig (CTLA-4 Ig) [24]. An alternative to the use of potentially toxic sub-lethal irradiation and myeloablative agents, or cumbersome high doses of bone marrow, is the administration of multiple doses under the cover of a single agent such as anti-CD40L antibody [28]. Mice treated with this protocol also developed robust haematopoietic chimaerism and donor specific tolerance to fully MHC mismatched skin grafts [28]. By extending these observations, Larsen *et al.* were able to develop a protocol using busulfan, an alkylating agent that preferentially depletes early haematopoietic stem cells, in conjunction with bone marrow administration and simultaneous blockade of the CD40 and CD28 co stimulatory pathways (using anti-CD40L and CTLA-4 Ig respectively) to establish for titratable levels of haematopoietic chimaerism that could result in donor specific tolerance in a mouse skin allograft model [14].

Recent clinical data suggest that long lasting mixed chimaerism may not be essential for the induction of tolerance to donor alloantigens. In recent patient studies, the majority of recipients of HLA-haploidentical stem cell transplantation with nonmyeloablative conditioning and immunosuppressive GVHD prophylaxis were able to achieve 100% donor cell engraftment and maintained graft function without the onset of GVHD with a mismatch of 2–3 HLA antigens [29,30].

Data from the experimental rat model have also demonstrated the possibility that pretransplant conditioning of the host may not always be required to attain the long-term survival of donor derived cells. Injection of rat embryonic stem like cells (RESCs) via the portal vein was found to result in a state of mixed chimaerism in which 5–8% of donor cells resided in the white blood cell population of recipient rats. Furthermore, rats with surviving RESCs were able to accept cardiac allografts permanently in a donor alloantigen specific manner [31]. One question arising from this study is how the RESCs survive in an allogeneic environment without host conditioning. Fas ligand (FasL) expressed on RESCs may render peripheral blood lymphocytes susceptible to apoptosis, a hypothesis supported by *in vitro* studies using RESCs. Thus, it may be possible to harness FasL dependent mechanism to avoid rejection when donor cells are injected into a nonmyeloablated host [31].

In a recent work, multipotent bone-marrow derived stromal cells, or mesenchymal stem cells (MSC) have been shown to possess an immunoregulatory capacity, at high doses, by suppressing the activation and proliferation of both naïve and memory T cells *in vitro* [32–34]. Furthermore, MSCs have shown promise in the facilitation of haematopoietic stem cell engraftment and attenuation of GVHD in limited clinical trials and have even been reported to upregulate regulatory T-cell subsets (discussed below) [35,36]. Although promising *in vitro*, MSCs have been quite controversial *in vivo*. Recent data on the effects of MSCs on immunomodulation in the rat allogeneic cardiac transplant transplant model suggest that MSCs may suppress T-cell proliferative responses *in vitro*; however, translation *in vivo* was not achieved as allograft survival was not prolonged and rejection responses were, in fact, accelerated [37]. More work to substantiate these findings in other experimental models is essential to progress for this approach to large animal models.

## Peripheral transplantation tolerance

Central deletion of auto or self reactive T cells in the thymus is a relatively incomplete process. Therefore, the immune system has developed additional strategies for regulating the functional capacity of T cells with potential autoreactivity that escape deletion in the thymus and emerge into the periphery. Peripheral tolerance is the term applied to these naturally arising mechanisms that lead to anergy, deletion or suppression of self reactive T cells in the periphery. Investigations in rodent models have sought to develop these mechanisms to obtain peripheral tolerance to alloantigens. Costimulation blockade is one approach that has been shown to induce peripheral tolerance to alloantigens and is discussed in the section below.

### Blockade of co stimulatory molecules

Blockade of co stimulatory molecules at the time of alloantigen recognition has been shown in experimental models to be a potential strategy for inducing peripheral tolerance. Engagement of B7.1/B7.2 (CD80/CD86 found on APCs) with CD28 (found on T cells) at the time of antigen recognition induces T cells to produce IL-2, a cytokine involved in their growth and proliferation [38]. Blockade of this pathway, *in vitro*, inhibits alloresponses and induces T-cell anergy [39]. In rodent models, there have been attempts to block signalling through the B7-CD28 pathway using a CTLA-4 Ig fusion protein (a soluble recombinant protein, which contains the extracellular domain of human CTLA-4 fused to human immunoglobulin Cγ chain). CTLA-4 (CD152), a molecule that is induced on activated T cells, is often referred to as a natural regulator of immune responsiveness. CTLA-4 binds to CD80 and CD86 with higher avidity than CD28 and can therefore compete with CD28 for binding to its ligands.

CTLA-4Ig has been used to treat recipients at the time of transplantation with promising results [40]. Interestingly, the most effective approaches reported in rodent models have combined CTLA-4Ig therapy with an infusion of donor alloantigen. When CTLA-4Ig was administered to mice treated with a donor specific transfusion (DST) cardiac allografts were found to survive indefinitely [41]. The beneficial effects of CTLA-4Ig have not been found in every experimental model examined, however. For example, the use of CTLA-4Ig monotherapy in primates has not been reported to be capable of inducing long-term graft survival [42]. However, Zheng *et al.* reported that treatment with CTLA-4Ig either pre or post-transplantation resulted in skin allograft rejection in mice pretreated with a tolerizing protocol of anti-CD40L/DST, which led the authors to conclude that signalling through CTLA-4 is required to achieve permanent graft acceptance [43].

CTLA-4-Ig therapy has been explored in clinical trials in solid organ and bone marrow transplantation. In the latter setting, donor bone marrow, which was mismatched with the recipient for one HLA haplotype, was cocultured with irradiated recipient cells for 36 h in the presence of CTLA-4Ig. Transfusion of these donor cells into the recipient led to a reduction in the frequency of donor specific alloreactive T cells and engraftment of the bone marrow. In addition, only three of 11 transplant recipients showed any evidence of GVHD thus suggesting that treatment of donor bone marrow *ex vivo* with CTLA-4Ig could reconstitute haematopoiesis *in vivo* with a reduced risk of GVHD [44]. Furthermore, recent evidence suggests that CTLA4-Ig is critical in the induction of chimaerism achieved in a model of mouse bone marrow transplantation and is independent of indolamine 2,3 dioxygenase production [45].

Experiment models using CTLA-4Ig laid the groundwork for further pharmacotherapeutic developments targeted at the B7:CD28/CTLA4 pathway. The most promising of these developments was the introduction of Belatacept (LEA29Y), a derivative of CTLA-4Ig [46,47]. Belatacept differs from CTLA-4Ig by two amino acid sequences, which confer a twofold greater ligation capacity to CD80 and CD86. This increase in avidity allows for an overall increase in the suppression of T-cell activation *in vitro* when compared with CTLA-4Ig [46]. Originally in nonhuman primate studies, Belatacept was found to prolong renal allograft survival and inhibit donor-specific alloantibody production both alone and in combination with other traditionally used immunosuppressive regimens [46]. These and other findings allowed for the translation of Balatacept to renal transplant patients in the clinics. To date, results of phase 2 trials (up to 12 months post-transplantation) comparing Belatacept to cyclosporine in partially randomized studies across 22 centers in North America and Europe of over 200 patients suggest that Belatacept is not inferior to cyclosporine in its ability to prevent acute rejection. In fact, the results of this trial, so far, suggest that patients with Belatacept-based therapy had improved renal function, decreased calcineurin-related toxicity, and no thrombo-embolic complications because of the exclusion of the CD154 pathway [48]. Additionally, recent experiments in nonhuman primates using neonatal porcine islet grafts have shown long-term xenograft survival under the cover of CD28–CD154 blockade with maintenance immunosuppression of sirolimus and belatacept [49]. Although promising, further trials and vigilant follow-up is necessary to assess accurately the efficacy of these new therapeutic regimens incorporating belatacept.

A second co stimulatory pathway of importance is the CD40 (found on APCs)/CD40L (CD154) (found on T cells) pathway [40], which plays a pivotal role in the development of CD4^+^ T-cell responses [50]. Attempts to induce tolerance by blockade of this pathway using monoclonal antibodies either alone or in combination with donor antigen have been successful in some experimental donor-recipient combinations [43,51]. For example, in a mouse skin allograft model, treatment with anti-CD40L mAb and DST was found to lead to prolonged survival of skin grafts by inducing the deletion of alloreactive CD8^+^ T cells [51]. In addition, prolonged survival of skin grafts could be abrogated by treating recipients with anti-CTLA-4 mAb further confirming that signalling through CTLA-4 was required for prolonged graft survival [43,51].

In general, CD4^+^ T cells are more susceptible to co stimulatory blockade than CD8^+^ T cells; therefore, in some rodent models the allograft survival can only occur if the co stimulatory molecule blocking agent is used in conjunction with an agent that depleted CD8^+^ T cells [52–54]. In a study investigating both the CD40L and LFA-1 pathways in the quest for transplantation tolerance, it was demonstrated in a mouse model that concomitant blockade of CD40L and LFA-1 through the use of monoclonal antibodies led to robust dominant tolerance to pancreatic islet grafts, whereas targeting these co stimulatory pathways individually was only partially effective for the induction of long-term graft survival [55].

Long-term acceptance of cardiac, renal and islet allografts in several murine and nonhuman primate models was achieved with CD40 blockade using anti-CD154 monoclonal antibody as monotherapy or in conjunction with CD28 blockade [40,56–60]. However, the so-called tolerant states generated by anti-CD154 therapy alone have been shown to disappear when therapy is withdrawn, leading to rejection. Even with CD28 blockade, anti-CD154 therapy must be sustained to promote permanent engraftment of cardiac or islet grafts [59,61,62]. Further, induced tolerant states in rodents tend to be more robust when anti-CD154 therapy is combined with donor antigens before transplantation tolerance is induced [57–59]. Although promising results were reported in experimental models, anti-CD154 therapy was found to have the unexpected complication of thrombogenesis. Some reports suggest that CD154 acts to stabilize thrombi while others implicate CD154 in platelet activation [63]. Whatever the role that CD154 may play in transplantation tolerance, it is clear that this molecule acts via independent pathways in a variety of cascades unrelated to tolerance induction [64]. Additionally, recent work by Larsen *et al.* has investigated the agonistic role that human chimaeric antibodies to CD40 (Chi220) have in abrogating immune responses. In these nonhuman primate models, the use of Chi220 alone was not impressive in the prolongation of renal and islet allografts; however, when combined with belatacept therapy allograft survival was markedly improved. These data suggest that future investigations of tolerance induction via costimulation blockade are necessary [65,66].

Some of the newly discovered co stimulatory molecules may also be targets for transplantation tolerance induction. In a mouse cardiac allograft model, mice deficient in the induced co stimulatory molecule – ICOS (ICOS^−/−^) showed prolonged allograft survival. Additionally, blockade of ICOS using an anti-ICOS antibody in conjunction with cyclosporine administration led to sustained allograft survival without the development of transplant arteriosclerosis [67,68]. Manipulation of the co stimulatory pathway consisting of the constitutive activated T-cell marker- herpes virus entry mediator (HVEM) and its ligand LIGHT found on APCs has also shown promise with regard to allograft survival. LIGHT^−/−^ mice treated with cyclosporine showed prolonged survival of cardiac allografts, decreased intragraft expression of IFN-γ and IFN-γ induced chemokine inducible protein -10 [69]. As the T cell–T-cell mediated LIGHT-HVEM co stimulatory pathway is an important component of the immune response, strategies to block or alter this pathway may contribute to induction of tolerance. A recently defined CD28 homologue and co stimulatory molecule, programmed death-1 (PD-1) and its ligands PDL-1 and PDL-2 (homologue of B7) are also of therapeutic interest. In a cardiac allograft model, CD28^−/−^ mice treated with a PD-L1 Ig fusion protein showed prolonged allograft survival, and in some cases permanent survival [70]. Treatment with PD-L1 Ig also prevented the development of transplant vasculopathy and prevented islet allograft destruction after anti-CD40L therapy, showing great promise [67,71].

## T-cell immunoregulation

Regulatory mechanisms in both the innate and adaptive immune systems contribute to the overall outcome after transplantation with T-cell mediated immunoregulation playing a key role for inducing and maintaining peripheral tolerance *in vivo*. In both rodent and human systems, there is an emerging consensus that donor reactive immunoregulatory activity can be enriched within CD4^+^ T cells.

The phenomenon of T-cell-mediated regulation in transplantation tolerance is not new, but recently, a number of interesting findings have brought it back into the limelight. Harnessing the capability of these suppressor cells to regulate immune responses to not only self molecules but also to foreign antigen is important as a potential therapy in transplantation. The ability of these regulatory T cells to induce unresponsiveness to alloantigen *in vivo*, in the absence of chronic immunosuppression, may inhibit the immune-mediated processes that lead to long-term graft failure.

### Infectious tolerance

**Figure 1:**
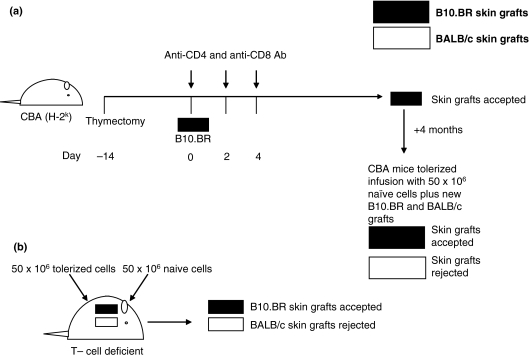
Demonstration of infectious tolerance in a mouse model. (a) Thymectomized CBA mice were transplanted with B10.BR skin grafts and given a tolerizing protocol of anti-CD4 and anti-CD8 antibodies. Four months later, infusion with 50 million naive splenocytes and transplantation of a new B10.BR skin graft was unable to break donor specific tolerance. However, tolerance could be broken if T cells in tolerant mice were depleted of CD4 T cells 7 weeks prior to transplantation of the second skin graft. (b) Fifty million spleen cells from tolerant and naive mice were adoptively transferred into T-cell deficient mice that were grafted with a B10.BR or BALB/c (third party skin). Cells from tolerant mice were able to suppress skin graft in a donor specific manner as BALB/c skin grafts were rejected.

Infectious tolerance is a process of peripheral immunoregulation, which is mediated by CD4^+^ T cells and results in the suppression of a primary or secondary immune response by disabling effector CD4^+^ T cells and converting them into regulatory T cells [72]. The concept of infectious tolerance was originally described by Medawar [1] and subsequently demonstrated by Qin *et al.* [73] in a model where adult thymectomized mice were tolerized with a cocktail of nondepleting anti-CD4 and anti-CD8 antibodies and accepted minor histocompatibility mismatched skin grafts. Infusion of naive syngeneic splenocytes and grafting with fresh skin grafts 4 months later was unable to break the tolerant status of these animals, and it was proposed that the T cells from tolerized mice were able to guide the naive effector cells into a tolerant state, rendering the tolerance achieved infectious (Fig. 1). The phenomenon of infectious tolerance is not exclusive to anti-CD4 therapy. Targeting other T-cell molecules as well, such as CD154, at the time of antigen recognition has also been shown to promote the development of infectious tolerance [74].

### CD25^+^ CD4^+^ regulatory T cells

As previously discussed, many autoreactive cells are deleted centrally in the thymus; however, some manage to escape. When these self reactive T cells emerge into the periphery, they have the capacity to respond to self peptides presented by self MHC molecules and therefore have the capacity to trigger the onset of autoimmune diseases. To prevent the development of these autoimmune diseases, the immune system must maintain a state of tolerance and active regulation of self-reactive leukocytes [75]. Prevention of autoimmunity has been described by ‘active’ mechanisms of tolerance that utilize a unique subset of T cells with regulatory function [76,77]. Suppressor or regulatory T cells have been implicated as a key factor in the active induction and maintenance of unresponsiveness to donor alloantigen *in vivo*, a characteristic that may prove to be crucial in the development of strategies to induce transplant tolerance [78].

Sakaguchi *et al.* were among the first to demonstrate that CD25 (IL-2R alpha) expression could be used as a tool to enrich a sub population of CD4^+^ T cells, which demonstrated powerful regulatory activity [76]. CD25 is constitutively found on approximately 10% of peripheral CD4^+^ T cells and <1% of peripheral CD8^+^ T cells. Although many studies within the last decade have demonstrated that CD25^+^ CD4^+^ T cells can mediate tolerance to self antigens [76,79–84], more recently, CD25^+^ CD4^+^ T cells have also been found to regulate responses to alloantigens.

**Figure 2:**
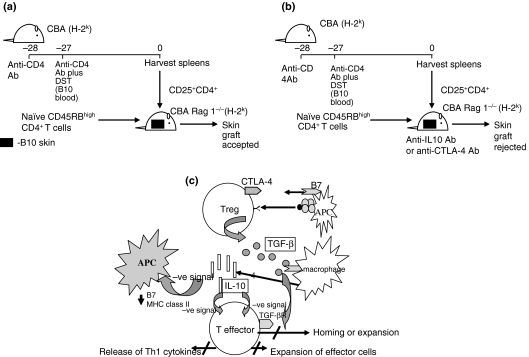
Demonstration of immunoregulation by CD25^+^ CD4^+^ T cells and proposed mechanism of action. (a) CD25^+^ CD4^+^ T cells isolated from CBA mice pretreated with anti-CD4 antibody plus DST are able to prevent B10 skin allograft rejection mediated by CD45RB^high^ CD4^+^ effector T cells. (b) Regulation mediated by CD25^+^ CD4^+^ T cells isolated from anti-CD4 antibody/DST treated mice is abrogated if recipient mice are administered an anti-IL-10 or anti-CTLA-4 antibody at the time of cell transfer (and weekly thereafter). (c) Crosslinking of CTLA-4 on regulatory T cells may lead to production of TGF-β which could bind to TGF-β receptors present on effector cells and prevent these cells expanding or homing to the graft. Alternatively, TGF-β may enhance the ability of macrophages to produce IL-10 which could deliver a negative signal to effector cells and prevent expansion or release of Th1 cytokines. Il-10 may also inhibit the function of APCs by down regulating B7 and MHC class II molecules.

Work from our own laboratory has revealed that CD25^+^ CD4^+^ T cells capable of regulating responses to alloantigens *in vivo* (Fig. 2), can be isolated from mice pretreated with a donor alloantigen, in the form of a specific transfusion (DST), under the cover of a nondepleting anti-CD4 monoclonal antibody in both the induction and maintenance phases of unresponsiveness [85,86]. In a model of bone marrow transplantation, Taylor *et al.* have demonstrated that CD25^+^ CD4^+^ regulatory T cells do not mediate GVHD and are essential for tolerance induction via co stimulatory blockade [87].

Such alloantigen specific CD25^+^ CD4^+^ regulatory T cells are able to prevent skin graft rejection initiated by not only CD4^+^ [85,88] but also CD8^+^ T cells [89], clearly indicating that these cells have the potential to control T-cell mediated rejection at multiple levels. Both our own studies [89] and those of Lin *et al.* [90] have explored the mechanisms by which CD25^+^ CD4^+^ regulatory T cells modulate CD8^+^ T-cell mediated rejection using Tg CD8^+^ T cells adoptively transferred into tolerant mice. CD8^+^ T-cell expansion was found not to be impaired; however, effector functions of CD8^+^ T cells were prevented from developing.

Graca *et al.* established that both CD25^+^ CD4^+^ T cells isolated from both naïve and tolerant mice could prevent the rejection of skin grafts mismatched for minor alloantigens [91]. CD25^+^ CD4^+^ T cells isolated from naive CBA mice were found to prevent rejection of B10.BR skin grafts when adoptively transferred into T-cell depleted recipients along with naive unsorted cells. However, the number of naive CD25^+^ CD4^+^ T cells required to regulate skin allograft rejection was at least 10-fold higher than CD25^+^ CD4^+^ T cells obtained from tolerant mice that had previously been exposed to the donor minor alloantigen. A similar observation was reported by Chen *et al.* in a rat model [92]. Data from our laboratory have shown that CD25^+^ CD4^+^ T cells isolated from naive mice adoptively transferred at equivalent cell doses as CD25^+^ CD4^+^ T cell from anti-CD4 (YTS177)/DST tolerized mice were unable to prevent rejection of fully MHC mismatched skin allografts [86]. Further information is required about the frequency of T cells capable of regulating responses to alloantigens that are present in naïve mice.

At present, a definitive marker to enrich regulatory cells is under active investigation. Other populations of regulatory cells clearly exist, including CD8^+^, CD8^+^ CD28^−^, TCR^+^ CD4^−^ CD8^−^ (‘double negative’) and natural killer T cells, thus CD25^+^ CD4^+^ cells may only represent one subset. The context in which the regulatory activity arises may impact the phenotypic and functional characteristics the regulatory populations possess. For example, in our system, CD25^−^ CD4^+^ T cells isolated from anti-CD4 antibody/DST treated mice were unable to prevent skin allograft rejection in our studies [85,86], whereas in other models, CD25^−^ CD4^+^ T cells from tolerant mice were able to prevent skin graft rejection mediated by unsorted spleen cells. This latter observation has been supported by data from Chiffoleau *et al.* demonstrated that tolerance could be transferred by thymic and splenic CD25^+^ CD4^+^ T cells, but in 50% of the cases, this transferable tolerance was mediated by splenic CD25^−^ CD4^+^ T cells [93]. Taken together, these findings suggest that CD25, although a useful marker, may not be an effective way of identifying regulatory T cells in all situations. Furthermore, reports describing distinct subsets of T cells (Tr1) with IL-10 dependent suppressive capacity are distinct to CD25^+^ CD4^+^ T reg in their low levels of CD25 expression [94].

The mechanisms by which CD25^+^ CD4^+^ regulatory T cells control responses to alloantigens are still under investigation. Recently, cytokines have been found to play an important role in mediating suppression in some systems *in vivo*. IL-10 and TGF-β play key roles in the suppressive activity of alloantigen specific CD25^+^ CD4^+^ regulatory T cells [86]. As depicted in Fig. 2, Tregs isolated from recipients pretreated with an anti-CD4/DST tolerizing protocol and co-transferred with naïve effector cells into T-cell deficient mice failed to prevent skin graft rejection when treated with an anti-IL-10 receptor antibody at the time of cell transfer and weekly thereafter, whereas in the absence of anti-IL-10R antibody treatment, all grafts were accepted [86] (Fig. 2). These findings also support data obtained in a mouse colitis model [84]. Similarly, TGF-β1 mRNA was expressed at high levels in accepted cardiac allografts from DST treated rats [95]. Moreover, neutralization of TGF-β*in vivo* from day 0 to day 4 post-transplantation abrogated tolerance, as six of six animals rejected their cardiac allografts.

In addition to cytokines, cell associated molecules have been found to be involved in immunoregulation in some situations. CD25^+^ CD4^+^ T cells have been shown in mice to express constitutively surface and cytoplasmic CTLA-4 (CD152) [80]. Although CTLA-4 functions at the cell surface, it is thought to be primarily stored intracellularly where it continuously cycles to and from the cell surface [96]. Data from our laboratory have shown that the population of CD25^+^ CD4^+^ T cells, which suppress skin allograft rejection are also dependent on signalling through CTLA-4, as blockade of CTLA-4 with anti-CTLA-4 antibody led to acute rejection of skin allografts [86].

The interplay among IL-10, TGF-β and CTLA-4 in the suppression of alloresponses is still not fully understood. Recent data have demonstrated a link between IL-10 and TGF-β, with IL-10 enhancing the expression of TGF-β receptor on activated and resting cells [97]. As cross linking of CTLA-4 has been shown to induce the production of TGF-β in one system [98], it is possible that a common mechanism of action may link CTLA-4 and IL-10 (Fig. 2).

An increasing list of molecules have found to be expressed by T cells with regulatory activity, including cell surface molecules such as CD62, CD103 and GITR (see below) and the transcription factor Foxp3. CD25^+^ CD4^+^ regulatory T cells in the thymus and periphery have been found to express the glucocorticoid induced tumor necrosis factor receptor (GITR) [99,100]. Signalling through this receptor (following treatment with the monoclonal antibody DTA-1) abrogated natural immune regulation and induce autoimmunity in normal mice [100]. As the authors hypothesize that GITR may play a role in immunoregulatory activity mediated by CD25^+^ CD4^+^ T cells, we might suggest that this molecule or its ligand could be of therapeutic interest in the generation of tolerance to both self and alloantigens. Investigations into the role of signalling through GITR in the induction and maintenance phases of tolerance have shown that their pathways may play a differential role, abrogating the induction of unresponsiveness but not affecting immunoregulation once it is established [101].

Overall, the models presented may have to be revisited, in the light of recent theories regarding the impact that memory T cells (Tm) may have on the induction of tolerance. The concept of ‘heterologous immunity’ proposed by Larsen’s group refers to chronic immunologic activation by various environmental stimuli leading to a population of Tm which can cross react with alloantigens. These Tm seem to be resistant to conventional induction therapies and may prove to be a hurdle for newer therapeutic approaches [102,103]. Although newer protocols involving central and peripheral tolerance may control naïve populations of effector cells, T cells that acquire immunologic memory are unique in phenotype and function when compared with their naïve counterparts. Specifically, Tm have a decreased threshold of activation and proliferation and also exhibit the ability to proliferate homeostatically, rendering this population difficult to contain with current experimental tolerance protocols [103]. Moreover, the benefits of tolerance induction may be broken after transplantation in the face of chronic infection, as suggested by certain experimental models [104,105]. Thus, through the use of more complex rodent models, it may be possible to develop experimental scenarios that more accurately reflect the distinct environments encountered when attempting to manipulate the immune response to a transplant in humans.

## Concluding remarks

It has become increasingly clear that research in experimental models has allowed for greater insight into the mechanisms of transplantation tolerance. These models have also established the principles in which new therapeutic approaches can be devised to enable robust tolerance to alloantigens to be achieved for the future of clinical transplantation (Table 1). Great care must be taken when trying to translate data from laboratory models to clinical application. Developing rodent models such that they replicate more accurately the distinct elements of the immune microenvironment that is present in humans is important to ensure that the findings in rodent models are more robust. Nevertheless, data derived from animal models can be extremely useful in directing the next phases of research required to develop novel therapeutic strategies for clinical application. Moreover, questions that arise as a result of a clinical investigation can often be explored very effectively by returning to rodent models to design new experiments that will provide clues as to how to approach the clinical problem. For example, the effects of current immunosuppressive regimens on tolerance inducing strategies could be explored initially through carefully designed experiments in rodent models of transplantation tolerance. Controlled clinical trials developed based on proof of concept and mechanistic studies in experimental models can yield promising results [106–111].

**Table 1:** Summary of strategies to induce transplantation tolerance in rodent models.

Type of tolerance	Rodent model	Strategy	Suggested mechanism of action	Reference
Central	Mouse islet allograft model	Intrathymic injection ofalloantigen	Deletion of alloreactive T cells	[4,8]
	Mouse cardiac allograft model	Intrathymic injection ofalloantigen	Deletion of alloreactive T cells	[5]
	Rat islet and cardiac allograftmodels	Intrathymic injection ofClass I peptide	Suppression of donor reactive CTLs	[7]
	Mouse skin allograft model	Bone marrow infusionand co stimulatoryblockade	Mixed chimerism	[24,28]
	Mouse skin allograft model	Bone marrow infusion,co stimulatory blockadeplus busulfan	Mixed chimerism	[14]
	Rat cardiac allograft model	Stem cell infusion viaportal vein	Mixed chimerism	[35]
Peripheral	Mouse skin allograft model	Tolerizingprotocol- thymectomy,anti-CD4 and anti-CD8 Ab	Infectious tolerance-alloreactiveT cells disabled	[43–45]
	Mouse skin allograft model	Generation and adoptivetransfer of CD25^+^ CD4^+^regulatory T cells	Suppression of alloreactiveT cells- involvement of IL-10and CTLA-4	[59,72,84,94]
	Mouse cardiac allograft model	Co stimulatory blockade	Prevents optimal T-cell activation/proliferation- alloreactive T cellsanergized and can undergoapoptosis	[83,93,101,103–105]
	Mouse skin allograft model	Co stimulatory blockade	As above	[95,99]
	Mouse islet allograft model	Co stimulatory blockade	As above	[64,102]

Furthermore, it is imperative to remember that manipulation of one aspect of the immune response may have a deleterious effect on other important immune pathways. Although the generation and expansion of CD25^+^ CD4^+^ T cells may be a strategy to induce donor specific transplantation tolerance, it has been well documented in a mouse tumour model that CD25^+^ regulatory T cells suppress tumour specific responses, leading to tumour growth. It is only when this subset is eliminated that tumour immunity can be restored [112–114]. Conversely, elimination of the CD25 subset to re-establish responses to tumour antigens has also been shown to lead to autoimmune destruction of melanocytes [115].

So what is the future direction of research into transplantation tolerance using experimental models? The identification of new co stimulatory pathways and the current interest in chemokines and their receptors may offer new targets for immune intervention that need to be fully explored in experimental models before such strategies can be considered and selected appropriately for further development. Although there is much information regarding the role regulatory T cells play in suppressing responses to self antigens, more research is needed to examine the role these cells play in regulating responses to alloantigens. One aim of transplant immunologists is to determine definitive markers of transplantation tolerance. Studies in animal systems may enable this goal to be achieved.

## References

[1] Billingham RE, Brent L, Medawar PB (1953). Actively acquired tolerance of foreign cells. Nature.

[2] Nankivell BJ, Borrows RJ, Fung CL, O'Connell PJ, Allen RD, Chapman JR (2003). The natural history of chronic allograft nephropathy. N Engl J Med.

[3] Wiesner RH, Menon KV (2001). Late hepatic allograft dysfunction. Liver Transpl.

[4] Posselt AM, Barker CF, Tomaszewski JE, Markmann JF, Choti MA, Naji A (1990). Induction of donor-specific unresponsiveness by intrathymic islet transplantation. Science.

[5] Jones ND, Fluck NC, Roelen DL, Mellor AL, Morris PJ, Wood KJ (1997). Deletion of alloantigen-reactive thymocytes as a mechanism of adult tolerance induction following intrathymic antigen administration. Eur J Immunol.

[6] Jones ND, Fluck NC, Mellor AL, Morris PJ, Wood KJ (1998). The induction of transplantation tolerance by intrathymic (i.t.) delivery of alloantigen: a critical relationship between i.t. deletion, thymic export of new T cells and the timing of transplantation. Int Immunol.

[7] Oluwole SF, Chowdhury NC, Ingram M, Garrovillo M, Jin MX, Agrawal S (1999). Mechanism of acquired thymic tolerance induced by a single major histocompatibility complex class I peptide with the dominant epitope: differential analysis of regulatory cytokines in the lymphoid and intragraft compartments. Transplantation.

[8] Turvey SE, Hara M, Morris PJ, Wood KJ (1999). Mechanisms of tolerance induction after intrathymic islet injection: determination of the fate of alloreactive thymocytes. Transplantation.

[9] Remuzzi G, Ferrazzi P, Bontempelli M (1995). Preliminary results of intrathymic injection of donor cells to prevent acute rejection in human heart transplantation. J Am Soc Nephrol.

[10] Ildstad ST, Sachs DH (1984). Reconstitution with syngeneic plus allogeneic or xenogeneic bone marrow leads to specific acceptance of allografts or xenografts. Nature.

[11] Sharabi Y, Sachs DH (1989). Mixed chimerism and permanent specific transplantation tolerance induced by a nonlethal preparative regimen. J Exp Med.

[12] Gianello PR, Fishbein JM, Rosengard BR (1995). Tolerance to class I-disparate renal allografts in miniature swine. Maintenance of tolerance despite induction of specific antidonor CTL responses. Transplantation.

[13] Cobbold SP, Adams E, Marshall SE, Davies JD, Waldmann H (1996). Mechanisms of peripheral tolerance and suppression induced by monoclonal antibodies to CD4 and CD8. Immunol Rev.

[14] Adams AB, Durham MM, Kean L (2001). Costimulation blockade, busulfan, and bone marrow promote titratable macrochimerism, induce transplantation tolerance, and correct genetic hemoglobinopathies with minimal myelosuppression. J Immunol.

[15] Myburgh JA, Smit JA, Stark JH, Browde S (1984). Total lymphoid irradiation in kidney and liver transplantation in the baboon: prolonged graft survival and alterations in T cell subsets with low cumulative dose regimens. J Immunol.

[16] Buhler LH, Spitzer TR, Sykes M (2002). Induction of kidney allograft tolerance after transient lymphohematopoietic chimerism in patients with multiple myeloma and end-stage renal disease. Transplantation.

[17] Spitzer TR, Delmonico F, Tolkoff-Rubin N (1999). Combined histocompatibility leukocyte antigen-matched donor bone marrow and renal transplantation for multiple myeloma with end stage renal disease: the induction of allograft tolerance through mixed lymphohematopoietic chimerism. Transplantation.

[18] Sykes M, Sachs DH (1988). Mixed allogeneic chimerism as an approach to transplantation tolerance. Immunol Today.

[19] Wekerle T, Sykes M (2004). Induction of tolerance. Surgery.

[20] Manilay JO, Pearson DA, Sergio JJ, Swenson KG, Sykes M (1998). Intrathymic deletion of alloreactive T cells in mixed bone marrow chimeras prepared with a nonmyeloablative conditioning regimen. Transplantation.

[21] Tomita Y, Khan A, Sykes M (1994). Role of intrathymic clonal deletion and peripheral anergy in transplantation tolerance induced by bone marrow transplantation in mice conditioned with a nonmyeloablative regimen. J Immunol.

[22] Guo Z, Wang J, Dong Y (2003). Long-term survival of intestinal allografts induced by costimulation blockade, busulfan and donor bone marrow infusion. Am J Transplant.

[23] Wekerle T, Sayegh MH, Hill J (1998). Extrathymic T cell deletion and allogeneic stem cell engraftment induced with costimulatory blockade is followed by central T cell tolerance. J Exp Med.

[24] Wekerle T, Kurtz J, Ito H (2000). Allogeneic bone marrow transplantation with co-stimulatory blockade induces macrochimerism and tolerance without cytoreductive host treatment. Nat Med.

[25] Huang CA, Fuchimoto Y, Scheier-Dolberg R, Murphy MC, Neville DM, Sachs DH (2000). Stable mixed chimerism and tolerance using a nonmyeloablative preparative regimen in a large-animal model. J Clin Invest.

[26] Luo B, Chan WF, Shapiro AM, Anderson CC (2007). Non-myeloablative mixed chimerism approaches and tolerance, a split decision. Eur J Immunol.

[27] Ildstad ST, Wren SM, Bluestone JA, Barbieri SA, Sachs DH (1985). Characterization of mixed allogeneic chimeras. Immunocompetence, in vitro reactivity, and genetic specificity of tolerance. J Exp Med.

[28] Durham MM, Bingaman AW, Adams AB (2000). Cutting edge: administration of anti-CD40 ligand and donor bone marrow leads to hemopoietic chimerism and donor-specific tolerance without cytoreductive conditioning. J Immunol.

[29] Ogawa H, Ikegame K, Yoshihara S (2006). Unmanipulated HLA 2–3 antigen-mismatched (haploidentical) stem cell transplantation using nonmyeloablative conditioning. Biol Blood Marrow Transplant.

[30] Ikegame K, Kawakami M, Yamagami T (2005). HLA-haploidentical nonmyeloablative stem cell transplantation: induction to tolerance without passing through mixed chimaerism. Clin Lab Haematol.

[31] Fandrich F, Lin X, Chai GX (2002). Preimplantation-stage stem cells induce long-term allogeneic graft acceptance without supplementary host conditioning. Nat Med.

[32] Bolanos-Meade J, Vogelsang GB (2006). Mesenchymal stem cells and organ transplantation: current status and promising future. Transplantation.

[33] Le Blanc K, Ringden O (2005). Immunobiology of human mesenchymal stem cells and future use in hematopoietic stem cell transplantation. Biol Blood Marrow Transplant.

[34] Le Blanc K, Tammik L, Sundberg B, Haynesworth SE, Ringden O (2003). Mesenchymal stem cells inhibit and stimulate mixed lymphocyte cultures and mitogenic responses independently of the major histocompatibility complex. Scand J Immunol.

[35] Le Blanc K, Rasmusson I, Sundberg B (2004). Treatment of severe acute graft-versus-host disease with third party haploidentical mesenchymal stem cells. Lancet.

[36] Aggarwal S, Pittenger MF (2005). Human mesenchymal stem cells modulate allogeneic immune cell responses. Blood.

[37] Inoue S, Popp FC, Koehl GE (2006). Immunomodulatory effects of mesenchymal stem cells in a rat organ transplant model. Transplantation.

[38] Linsley PS, Brady W, Grosmaire L, Aruffo A, Damle NK, Ledbetter JA (1991). Binding of the B cell activation antigen B7 to CD28 costimulates T cell proliferation and interleukin 2 mRNA accumulation. J Exp Med.

[39] Schwartz RH (1992). Costimulation of T lymphocytes: the role of CD28, CTLA-4, and B7/BB1 in interleukin-2 production and immunotherapy. Cell.

[40] Larsen CP, Elwood ET, Alexander DZ (1996). Long-term acceptance of skin and cardiac allografts after blocking CD40 and CD28 pathways. Nature.

[41] Judge TA, Wu Z, Zheng XG, Sharpe AH, Sayegh MH, Turka LA (1999). The role of CD80, CD86, and CTLA4 in alloimmune responses and the induction of long-term allograft survival. J Immunol.

[42] Kirk AD, Harlan DM, Armstrong NN (1997). CTLA4-Ig and anti-CD40 ligand prevent renal allograft rejection in primates. Proc Natl Acad Sci U S A.

[43] Zheng XX, Markees TG, Hancock WW (1999). CTLA4 signals are required to optimally induce allograft tolerance with combined donor-specific transfusion and anti-CD154 monoclonal antibody treatment. J Immunol.

[44] Guinan EC, Boussiotis VA, Neuberg D (1999). Transplantation of anergic histoincompatible bone marrow allografts. N Engl J Med.

[45] Pree I, Bigenzahn S, Fuchs D (2007). CTLA4Ig promotes the induction of hematopoietic chimerism and tolerance independently of Indoleamine-2,3-dioxygenase. Transplantation..

[46] Larsen CP, Pearson TC, Adams AB (2005). Rational development of LEA29Y (belatacept), a high-affinity variant of CTLA4-Ig with potent immunosuppressive properties. Am J Transplant.

[47] Bluestone JA, St Clair EW, Turka LA (2006). CTLA4Ig: bridging the basic immunology with clinical application. Immunity.

[48] Vincenti F, Larsen C, Durrbach A (2005). Costimulation blockade with belatacept in renal transplantation. N Engl J Med.

[49] Cardona K, Korbutt GS, Milas Z (2006). Long-term survival of neonatal porcine islets in nonhuman primates by targeting costimulation pathways. Nat Med.

[50] van Essen D, Kikutani H, Gray D (1995). CD40 ligand-transduced co-stimulation of T cells in the development of helper function. Nature.

[51] Iwakoshi NN, Mordes JP, Markees TG, Phillips NE, Rossini AA, Greiner DL (2000). Treatment of allograft recipients with donor-specific transfusion and anti-CD154 antibody leads to deletion of alloreactive CD8+ T cells and prolonged graft survival in a CTLA4-dependent manner. J Immunol.

[52] Honey K, Cobbold SP, Waldmann H (1999). CD40 ligand blockade induces CD4+ T cell tolerance and linked suppression. J Immunol.

[53] Jones ND, Van Maurik A, Hara M (2000). CD40-CD40 ligand-independent activation of CD8+ T cells can trigger allograft rejection. J Immunol.

[54] Meng L, Guo Z, Kim O (2001). Blockade of the CD40 pathway fails to prevent CD8 T cell-mediated intestinal allograft rejection. Transplant Proc.

[55] Nicolls MR, Coulombe M, Beilke J, Gelhaus HC, Gill RG (2002). CD4-dependent generation of dominant transplantation tolerance induced by simultaneous perturbation of CD154 and LFA-1 pathways. J Immunol.

[56] Larsen CP, Alexander DZ, Hollenbaugh D (1996). CD40-gp39 interactions play a critical role during allograft rejection. Suppression of allograft rejection by blockade of the CD40-gp39 pathway. Transplantation.

[57] Markees TG, Phillips NE, Noelle RJ (1997). Prolonged survival of mouse skin allografts in recipients treated with donor splenocytes and antibody to CD40 ligand. Transplantation.

[58] Niimi M, Pearson TC, Larsen CP (1998). The role of the CD40 pathway in alloantigen-induced hyporesponsiveness in vivo. J Immunol.

[59] Parker DC, Greiner DL, Phillips NE (1995). Survival of mouse pancreatic islet allografts in recipients treated with allogeneic small lymphocytes and antibody to CD40 ligand. Proc Natl Acad Sci U S A.

[60] Malm H, Pahlman C, Veress B, Corbascio M, Ekberg H (2006). Combined costimulation blockade prevents rejection of allogeneic islets in mice. Scand J Immunol.

[61] Sho M, Sandner SE, Najafian N (2002). New insights into the interactions between T-cell costimulatory blockade and conventional immunosuppressive drugs. Ann Surg.

[62] Hancock WW, Sayegh MH, Zheng XG, Peach R, Linsley PS, Turka LA (1996). Costimulatory function and expression of CD40 ligand, CD80, and CD86 in vascularized murine cardiac allograft rejection. Proc Natl Acad Sci U S A.

[63] Andre P, Prasad KS, Denis CV (2002). CD40L stabilizes arterial thrombi by a beta3 integrin–dependent mechanism. Nat Med.

[64] Larsen CP, Knechtle SJ, Adams A, Pearson T, Kirk AD (2006). A new look at blockade of T-cell costimulation: a therapeutic strategy for long-term maintenance immunosuppression. Am J Transplant.

[65] Adams AB, Shirasugi N, Jones TR (2005). Development of a chimeric anti-CD40 monoclonal antibody that synergizes with LEA29Y to prolong islet allograft survival. J Immunol.

[66] Pearson TC, Trambley J, Odom K (2002). Anti-CD40 therapy extends renal allograft survival in rhesus macaques. Transplantation.

[67] Snanoudj R, de Preneuf H, Creput C (2006). Costimulation blockade and its possible future use in clinical transplantation. Transpl Int.

[68] Ozkaynak E, Gao W, Shemmeri N (2001). Importance of ICOS-B7RP-1 costimulation in acute and chronic allograft rejection. Nat Immunol.

[69] Ye Q, Fraser CC, Gao W (2002). Modulation of LIGHT-HVEM costimulation prolongs cardiac allograft survival. J Exp Med.

[70] Ozkaynak E, Wang L, Goodearl A (2002). Programmed death-1 targeting can promote allograft survival. J Immunol.

[71] Gao W, Demirci G, Strom TB, Li XC (2003). Stimulating PD-1-negative signals concurrent with blocking CD154 co-stimulation induces long-term islet allograft survival. Transplantation.

[72] Cobbold S, Waldmann H (1998). Infectious tolerance. Curr Opin Immunol.

[73] Qin S, Cobbold SP, Pope H (1993). ‘‘Infectious’’ transplantation tolerance. Science.

[74] Graca L, Honey K, Adams E, Cobbold SP, Waldmann H (2000). Cutting edge: anti-CD154 therapeutic antibodies induce infectious transplantation tolerance. J Immunol.

[75] Powrie F, Read S, Mottet C, Uhlig H, Maloy K (2003). Control of immune pathology by regulatory T cells. Novartis Found Symp.

[76] Sakaguchi S, Sakaguchi N, Asano M, Itoh M, Toda M (1995). Immunologic self-tolerance maintained by activated T cells expressing IL-2 receptor alpha-chains (CD25). Breakdown of a single mechanism of self-tolerance causes various autoimmune diseases. J Immunol.

[77] Sakaguchi S (2004). Naturally arising CD4+ regulatory T cells for immunologic self-tolerance and negative control of immune responses. Annu Rev Immunol.

[78] Wood KJ, Sakaguchi S (2003). Regulatory T cells in transplantation tolerance. Nat Rev Immunol.

[79] Takahashi T, Tagami T, Yamazaki S (2000). Immunologic self-tolerance maintained by CD25(+)CD4(+) regulatory T cells constitutively expressing cytotoxic T lymphocyte-associated antigen4. J Exp Med.

[80] Read S, Malmstrom V, Powrie F (2000). Cytotoxic T lymphocyte-associated antigen 4 plays an essential role in the function of CD25(+)CD4(+) regulatory cells that control intestinal inflammation. J Exp Med.

[81] Itoh M, Takahashi T, Sakaguchi N (1999). Thymus and autoimmunity: production of CD25+CD4+ naturally anergic and suppressive T cells as a key function of the thymus in maintaining immunologic self-tolerance. J Immunol.

[82] Asano M, Toda M, Sakaguchi N, Sakaguchi S (1996). Autoimmune disease as a consequence of developmental abnormality of a T cell subpopulation. J Exp Med.

[83] Suri-Payer E, Amar AZ, Thornton AM, Shevach EM (1998). CD4+CD25+ T cells inhibit both the induction and effector function of autoreactive T cells and represent a unique lineage of immunoregulatory cells. J Immunol.

[84] Annacker O, Pimenta-Araujo R, Burlen-Defranoux O, Barbosa TC, Cumano A, Bandeira A (2001). CD25+ CD4+ T cells regulate the expansion of peripheral CD4 T cells through the production of IL-10. J Immunol.

[85] Hara M, Kingsley CI, Niimi M (2001). IL-10 is required for regulatory T cells to mediate tolerance to alloantigens in vivo. J Immunol.

[86] Kingsley CI, Karim M, Bushell AR, Wood KJ (2002). CD25+ CD4+ regulatory Tcells prevent graft rejection: CTLA-4- and IL-10-dependent immunoregulation of alloresponses. J Immunol.

[87] Taylor PA, Noelle RJ, Blazar BR (2001). CD4(+)CD25(+)immune regulatory cells are required for induction of tolerance to alloantigen via costimulatory blockade. J Exp Med.

[88] Golshayan D, Jiang S, Tsang J, Garin MI, Mottet C, Lechler RI (2007). In vitro-expanded donor alloantigen-specific CD4+CD25+ regulatory T cells promote experimental transplantation tolerance. Blood.

[89] van Maurik A, Herber M, Wood KJ, Jones ND (2002). Cutting edge: CD4+CD25+ alloantigen-specific immunoregulatory cells that can prevent CD8+ T cell-mediated graft rejection: implications for anti-CD154 immunotherapy. J Immunol.

[90] Lin CY, Graca L, Cobbold SP, Waldmann H (2002). Dominant transplantation tolerance impairs CD8+ T cell function but not expansion. Nat Immunol.

[91] Graca L, Thompson S, Lin CY, Adams E, Cobbold SP, Waldmann H (2002). BothCD4(+)CD25(+)andCD4(+)CD25(-) regulatory cells mediate dominant transplantation tolerance. J Immunol.

[92] Chen J, Huoam C, Plain K, He XY, Hodgkinson SJ, Hall BM (2001). CD4 (+),CD25 (+) T cells as regulators of alloimmune responses. Transplant Proc.

[93] Chiffoleau E, Beriou G, Dutartre P, Usal C, Soulillou JP, Cuturi MC (2002). Role for thymic and splenic regulatory CD4+ T cells induced by donor dendritic cells in allograft tolerance by LF15-0195 treatment. J Immunol.

[94] Levings MK, Sangregorio R, Sartirana C (2002). Human CD25+CD4+ T suppressor cell clones produce transforming growth factor beta, but not interleukin 10, and are distinct from type 1 T regulatory cells. J Exp Med.

[95] Josien R, Douillard P, Guillot C (1998). A critical role for transforming growth factor-beta in donor transfusion-induced allograft tolerance. J Clin Invest.

[96] Walunas TL, Lenschow DJ, Bakker CY (1994). CTLA-4 can function as a negative regulator of T cell activation. Immunity.

[97] Cottrez F, Groux H (2001). Regulation of TGF-beta response during T cell activation is modulated by IL-10. J Immunol.

[98] Chen W, Jin W, Wahl SM (1998). (CTLA-4) induces transforming growth factor beta (TGF-beta) production by murine CD4(+) T cells. J Exp Med.

[99] McHugh RS, Whitters MJ, Piccirillo CA (2002). CD4(+)CD25(+) immunoregulatory T cells: gene expression analysis reveals a functional role for the glucocorticoid-induced TNF receptor. Immunity.

[100] Shimizu J, Yamazaki S, Takahashi T, Ishida Y, Sakaguchi S (2002). Stimulation of CD25(+)CD4(+) regulatory T cells through GITR breaks immunological self-tolerance. Nat Immunol.

[101] Wood KJ, Ushigome H, Karim M, Bushell A, Hori S, Sakaguchi S (2003). Regulatory cells in transplantation. Novartis Found Symp.

[102] Trzonkowski P, Zilvetti M, Friend P, Wood KJ (2006). Recipient memory-like lymphocytes remain unresponsive to graft antigens after CAMPATH-1H induction with reduced maintenance immunosuppression. Transplantation.

[103] Adams AB, Pearson TC, Larsen CP (2003). Heterologous immunity: an overlooked barrier to tolerance. Immunol Rev.

[104] Williams MA, Onami TM, Adams AB (2002). Cutting edge: persistent viral infection prevents tolerance induction and escapes immune control following CD28/CD40 blockade-based regimen. J Immunol.

[105] Pantenburg B, Heinzel F, Das L, Heeger PS, Valujskikh A (2002). T cells primed by *Leishmania major* infection cross-react with alloantigens and alter the course of allograft rejection. J Immunol.

[106] Calne R, Moffatt SD, Friend PJ (1999). Campath IH allows low-dose cyclosporine monotherapy in 31 cadaveric renal allograft recipients. Transplantation.

[107] Wasch R, Reisser S, Hahn J (2000). Rapid achievement of complete donor chimerism and low regimen-related toxicity after reduced conditioning with fludarabine, carmustine, melphalan and allogeneic transplantation. Bone Marrow Transplant.

[108] Bolinger AM, Zangwill AB, Slattery JT (2001). Target dose adjustment of busulfan in pediatric patients undergoing bone marrow transplantation. Bone Marrow Transplant.

[109] Kottaridis PD, Milligan DW, Chopra R (2001). Invivo CAMPATH-1H prevents GvHD following nonmyeloablative stem-cell transplantation. Cytotherapy.

[110] Spitzer TR, Sykes M (2001). Treatment of renal-cell cancer by transplantation of allogeneic stem cells. N Engl J Med.

[111] Kobbe G, Schneider P, Aivado M (2002). Reliable engraftment, low toxicity, and durable remissions following allogeneic blood stem cell transplantation with minimal conditioning. Exp Hematol.

[112] Onizuka S, Tawara I, Shimizu J, Sakaguchi S, Fujita T, Nakayama E (1999). Tumor rejection by in vivo administration of anti-CD25 (interleukin-2 receptor alpha) monoclonal antibody. Cancer Res.

[113] Shimizu J, Yamazaki S, Sakaguchi S (1999). Induction of tumor immunity by removing CD25+CD4+ T cells: a common basis between tumor immunity and autoimmunity. J Immunol.

[114] Sutmuller RP, van Duivenvoorde LM, van Elsas A (2001). Synergism of cytotoxic T lymphocyte-associated antigen 4 blockade and depletion of CD25(+) regulatory T cells in antitumor therapy reveals alternative pathways for suppression of autoreactive cytotoxic T lymphocyte responses. J Exp Med.

[115] Jones E, Dahm-Vicker M, Simon AK (2002). Depletion of CD25+ regulatory cells results in suppression of melanoma growth and induction of autoreactivity in mice. Cancer Immun.

